# *Salmonella* Effector SpvB Disrupts Intestinal Epithelial Barrier Integrity for Bacterial Translocation

**DOI:** 10.3389/fcimb.2020.606541

**Published:** 2020-12-17

**Authors:** Lanqing Sun, Sidi Yang, Qifeng Deng, Kedi Dong, Yuanyuan Li, Shuyan Wu, Rui Huang

**Affiliations:** Department of Medical Microbiology, School of Biology and Basic Medical Sciences, Medical College of Soochow University, Suzhou, China

**Keywords:** *Salmonella*, SpvB, intestinal epithelial barrier, apical junctional complex, F-actin, protein kinase C

## Abstract

*Salmonella* are common enteric bacterial pathogens that infect both humans and animals. Intestinal epithelial barrier, formed by a single layer of epithelial cells and apical junctional complex (AJC), plays a crucial role in host defense against enteric pathogens to prevent bacterial translocation. However, the underlying mechanisms of intestinal epithelial barrier dysfunction caused by *Salmonella* are poorly understood. It is found that a locus termed *Salmonella* plasmid virulence (*spv*) gene exists extensively in clinically important *Salmonella* serovars. SpvB is a key effector encoded within this locus, and closely related to *Salmonella* pathogenicity such as interfering with autophagy and iron homeostasis. To investigate the interaction between SpvB and intestinal epithelial barrier and elucidate the underlying molecular mechanism, we used the typical foodborne disease agent *Salmonella enterica* serovar Typhimurium (*Salmonella typhimurium*) carrying *spvB* or not to construct infection models *in vivo* and *in vitro*. C57BL/6 mice were orally challenged with *S. typhimurium* wild-type strain SL1344 or *spvB*-deficient mutant strain SL1344-Δ*spvB*. Caco-2 cell monolayer model, as a widely used model to mimic the human intestinal epithelium *in vitro*, was infected with SL1344, SL1344-Δ*spvB*, or *spvB* complementary strain SL1344-c-Δ*spvB*, respectively. The results showed that SpvB enhanced bacterial pathogenicity during *S. typhimurium* infection *in vivo*, and contributed to intestinal epithelial barrier dysfunction in both infection systems. This SpvB-mediated barrier dysfunction was attributed to the cellular redistribution of Claudin-1, Occludin, and E-cadherin junctional proteins. Moreover, by using pharmacological inhibitors, we found that F-actin rearrangement and suppression of protein kinase C (PKC) signaling pathway were involved in SpvB-mediated barrier dysfunction. In conclusion, the study reveals the contribution of *Salmonella* effector SpvB to the dysfunction of intestinal epithelial barrier integrity, which facilitates bacterial translocation *via* the paracellular route to promote *Salmonella* systemic dissemination. Our findings broaden the understanding of host–pathogen interactions in salmonellosis, and provide new strategies for the therapy in limiting bacterial dissemination during infection.

## Introduction

*Salmonella enterica* (*S. enterica*) are facultative intracellular pathogens that can cause both localized and disseminated disease. Consisting of more than 2,600 different serovars, *S*. *enterica* can be divided into typhoidal and non-typhoidal *S*. *enterica* serovars according to species specificity and diverse clinical manifestations. Typhoidal *S*. *enterica* serovars are human host–restricted pathogens, and typically result in typhoid and paratyphoid fevers (collectively referred to as enteric fever). Whereas non-typhoidal *S*. *enterica* serovars, such as Typhimurium and Enteritidis (*Salmonella typhimurium* and *S. enteritidis*), are global causes of diarrheal diseases with life-threatening bacteremia occurring sometimes both in humans and animals (Zhang et al., 2018; Collaborators, 2019). Despite the different disease outcomes determined by conflicts between host and pathogen, all these *Salmonella* serovars have to overcome the same intestinal barrier in order to successfully colonize the host (Hume et al., 2017). In this study, we used *S. typhimurium* to study host–pathogen interactions in salmonellosis because it acts as a typical foodborne disease agent with a high mortality rate, which is widely used to establish infection models both *in vitro* and *in vivo* (Wotzka et al., 2017).

For strains belonging to clinically important serovars, e.g., *S. enteritidis*, *S. typhimurium*, *S. choleraesuis*, and *S. Dublin*, it is found an additional locus termed *Salmonella* plasmid virulence gene (*spv*) located within a highly homologous region contained on virulence plasmids, with *spvABCD* genes arranged in an operon positively regulated by the upstream *spvR* gene (Fabrega and Vila, 2013). Among them, *spvB* gene is extensively studied due to its distinct contribution for the virulence phenotype in mice (Guiney and Fierer, 2011). Our pervious findings have shown the function of SpvB on interfering with autophagy and iron homeostasis in immune cells like macrophages and epithelial cells like HeLa cells, as well as *in vivo* using mouse and zebrafish infection models (Chu et al., 2016; Li et al., 2016; Yang et al., 2019). However, the underlying mechanisms of *Salmonella* pathogenicity contributed by SpvB is still not well known. A more comprehensive study of the effector SpvB employed by *Salmonella* could help us further understand the interactions between host and pathogen in infectious disease.

The gastrointestinal tract is the primary route of *Salmonella* oral infection. An effective intestinal barrier consists of multiple layers, including the biological, the chemical, the immune and the epithelial barriers, to orchestrate intestinal homeostasis. Among this dynamic barrier, the intestinal epithelial barrier acts as a basic defense against pathogenic bacteria from intruding into the mucosa and cause infectious diseases (Barreau and Hugot, 2014). It is formed by a single layer of the intestinal epithelial cells (IECs) joined to each other by the junctional complexes. The tight junction (TJ) and the subjacent adherens junction (AJ) are collectively referred to as the apical junctional complex (AJC), which has a crucial role in the regulation of intestinal barrier function (Quiros and Nusrat, 2014). TJ is the most apical structure of AJC formed by transmembrane proteins such as Occludin, Claudin family members and junctional adhesion molecules. Similarly, AJ is positioned immediately below TJ composed of the transmembrane protein E-cadherin, linked to adaptor proteins and actin cytoskeleton. Controlled by signaling pathways such as protein kinase C (PKC) pathway and so on, these AJC transmembrane proteins separate the apical and basolateral cell surfaces to provide a barrier function, limit solute and water flow through the paracellular space, and prevent microbes from invasion. A growing number of studies have demonstrated that enteric pathogens very often disrupt AJC through the secretion of toxins thus altering bacterial translocation *via* the paracellular route (Boyle et al., 2006; Burkholder and Bhunia, 2010). However, the effect of factors encoded by *Salmonella*, especially by *Salmonella* virulence plasmids on the intestinal epithelial barrier needs to be elucidated. Since it is previously known that *Salmonella* effector SpvB could function on preventing actin polymerization (Tezcan-Merdol et al., 2001), which is of vital importance in maintenance of AJC, we focus on the interaction between SpvB and intestinal epithelial barrier to further described its role during *Salmonella* infection.

Here, we further investigated the function of the effector SpvB and proposed its contribution to intestinal barrier dysfunction during *Salmonella* infection. We found SpvB disrupted the intestinal epithelial barrier function and facilitated *Salmonella* dissemination through redistribution of AJC proteins, with the involvement of F-actin rearrangement and suppression of PKC signaling pathway. Our study provides a new insight into SpvB-mediated bacterial pathogenicity and broadens our understanding of host–pathogen interactions in salmonellosis.

## Materials and Methods

### Bacterial Strains and Growth Conditions

*S. typhimurium* wild-type strain SL1344, *spvB*-deficient mutant (SL1344-Δ*spvB*) and its complement (SL1344-c-Δ*spvB*) in plasmid pBAD/gIIIA were used in the study as previously described (Yang et al., 2019). All of the *Salmonella* strains were grown in Luria Bertani (LB) medium (Hangwei, Hangzhou, China) at 37°C with shaking overnight, then diluted 1:100 with fresh LB medium and cultured until the logarithmic phase was reached. LB medium was supplemented with 100 µg/ml ampicillin when appropriate.

### Mouse Experiments

C57BL/6 mice (6–8 weeks) obtained from the experimental animal center of Soochow university were fed ad libitum under specific-pathogen-free conditions. All animal procedures were approved by the Ethic Committee of Soochow University, Suzhou, China and were carried out in accordance with the Guidelines for the Care and Use of Research Animals established by Soochow University. For experiment, the mice were pretreated with streptomycin (0.1 ml of a 200 mg/ml solution in sterile water) intragastrically 24 h before inoculation with different *S. typhimurium* strains (100 μl containing 1 × 10^8^ colony forming units (CFUs)). The control mice received only PBS. Mice were euthanized 48 h p.i. using CO_2_ asphyxiation. For histopathology, tissue samples were harvested and fixed in 4% paraformaldehyde, processed according to standard procedures for paraffin embedding, sectioned at 5 μm, and stained with hematoxylin and eosin (H&E). Images of histopathologic changes were acquired using a Nikon Eclipse Ni-U fluorescence microscope with NIS-Elements. For enumerating bacterial CFUs in mouse organs, samples of liver, spleen, MLN and colon were weighed and homogenates in PBS containing 0.5% Tergitol (NP9; Sigma-Aldrich, St. Louis, MO, USA) and 0.5% fetal bovine serum (FBS; SFBS-M; BOVOGEN, East Keilor, VIC, AUS) were serially diluted in PBS and plated on *Salmonella*-*Shigella* plates. To enumerate bacteria in the mucus, epithelial cell and the lamina propria fractions from the colon tissue, a previously described protocol (Drolia et al., 2018) was used.

### Intestinal Permeability *In Vivo*

For analysis of intestinal permeability *in vivo*, the mice were orally administered with 12 mg/mouse 4 kDa FITC-dextran (46944; Sigma-Aldrich) 4 h prior to sacrifice. Serum was collected by cardiac puncture and fluorescence was measured (Em: 485 nm; Ex: 528 nm; Biotek, Synergy 2). The concentration of the FITC-dextran was calculated using a standard curve generated by serially diluting FITC-dextran in PBS.

### Cell Culture and Infection

The human colon carcinoma Caco-2 cell line and the human embryonic kidney 293T cell line were kindly provided by Professor Weiqi He and Professor Ying Xu respectively (Soochow University, Suzhou, China). Cells from 25 to 35 passages were cultured in Dulbecco’s Modified Eagle’s medium (DMEM) (SH30243.01B; Hyclone, South Logan, UT, USA) supplemented with 10% FBS and 1% Penicillin-Streptomycin Solution (C0222; Beyotime Biotechnology, Shanghai, China) at 37°C with 5% CO_2_. For infection, Bacteria were washed three times in PBS and resuspended in DMEM-FBS (10%) at a multiplicity of infection (MOI) of ~100. One hour later, infected cells were washed three times with PBS and replaced by DMEM-FBS (10%) containing 100 µg/ml amikacin to eliminate extracellular bacteria. For inhibition of actin polymerization, Caco-2 cells were pretreated with 2 µM Cytochalasin D (ab143484; abcam, Cambridge, MA, USA) for 1 h. For inhibition of PKC activation, Caco-2 cells were pretreated with 1 µM Bisindolylmaleimide I (S7208; Selleck, Houston, TX, USA) for 1 h.

### TEER, Bacterial Translocation and Intestinal Permeability *In Vitro*

To form cell monolayers, Caco-2 cells were grown on Transwell inserts (Corning-Costar, Corning, NY, USA) for up to 14 to 21 days. TEER was measured by Millicells Voltmeter (Millipore, Burlington, MA, USA) to monitor the monolayer integrity. A TEER value of at least 200 Ω/cm^2^ (± 10) was used as the basal value to monitor the monolayer integrity (Burkholder and Bhunia, 2010). For infection, Bacteria resuspended in DMEM-FBS (10%) were added to the apical side of the Transwell system, TEER of the monolayer was measure every 0.5 h. For bacterial translocation analysis, the liquid was collected from the basal well after 3 h incubation, and translocated bacteria were enumerated by plating (Burkholder and Bhunia, 2010). For analysis of intestinal permeability *in vitro*, 5 mg/ml 4 kDa FITC-dextran with bacteria resuspended in DMEM-FBS (10%) was added to the apical side of the Transwell system. After 3 h incubation, the liquid was collected from the basal well of Transwell system, and the concentration of the FITC-dextran was measured as described above.

### Bacterial Adhesion and Bacterial Invasion

Caco-2 cells were seeded in 12-well plates. For bacterial adhesion analysis, cells were washed with PBS after 0.5 h of infection (MOI, ~100), then lysed with 0.3% Triton X-100 (V900502; Sigma-Aldrich), and bacteria were enumerated by plating. For bacterial invasion analysis, after 0.5 h of infection (MOI, ~100), the cells were incubated for an additional 0.5 h in DMEM-FBS (10%) containing amikacin (100 µg/ml). Cells were then lysed with 0.3% Triton X-100, and the internalized bacteria were enumerated by plating (Lin et al., 2016).

### Western Blot Analysis

To extract the proteins from Caco-2 cells, cells were seeded in 6-well plates for 14 to 21 days. Following treatment, total protein from Caco-2 cells was extracted with RIPA Lysis Buffer (P0013B; Beyotime Biotechnology) containing the Protease and Phosphatase Inhibitor Cocktail (P1045; Beyotime Biotechnology). Detergent-insoluble (membrane) and detergent-soluble (cytosolic) proteins were isolated using a Membrane and Cytosol Protein Extraction Kit (P0033; Beyotime Biotechnology) according to the manufacturer’s instructions. To extract proteins from colon epithelial cells, the epithelial cell fraction from colon tissues were isolated as described previously (Bou Ghanem et al., 2012) and total protein was extracted with RIPA Lysis Buffer. The protein concentrations were determined by BCA assay (P0012; Beyotime Biotechnology) according to the manufacturer’s instructions. Proteins were separated on 10%–12% polyacrylamide SDS-PAGE gels and electro-transferred to polyvinylidene difluoride (PVDF) membrane (IPVH00010; Millipore). The membranes were then blocked in 5% nonfat dry milk in Tris-buffered saline (TBS) containing 0.1% Tween 20 (TBST) for 0.5 h. All of the primary antibodies were diluted in 5% bovine serum albumin (BSA) in TBST and incubated overnight at 4°C. Secondary antibodies (1:2000 in 5% BSA in TBST) were incubated for 1 h at room temperature. The blots were visualized with an ECL luminescence reagent (ma0186; Meilunbio, Dalian, China). Following primary antibodies were used: anti-Claudin-1 antibody (ab15098; abcam); anti-Occludin antibody (13409-1-AP; Proteintech, Rosemont, IL, USA); anti-E-cadherin antibody (14472; Cell Signaling Technology, Danvers, MA, USA); anti-phospho-PKC (pan) antibody (9371; Cell Signaling Technology); anti-PKC antibody (yt3752; Immunoway, Plano, TX, USA); anti-β-actin antibody (bs-0061R; Bioss, Beijing, China) and anti-GAPDH antibody (bs-10900R; Bioss).

### Immunofluorescence Staining

The mouse colon-tissue sections were fixed with 4% paraformaldehyde and embedded in paraffin. The tissues were 5 µm-thick sectioned, deparaffinized, and rehydrated for antigen retrieval. For antibody labeling in Caco-2 cells, the cells were fixed with 4% paraformaldehyde for 15 min. The tissue sections and Caco-2 cells were permeabilized and blocked with PBS containing 0.3% Triton X-100 and 3% BSA, then immunostained with specific antibodies by incubating overnight at 4°C. Following primary antibody incubation, slides were washed three times with PBS and incubated with Alexa Fluor 555 goat anti-rabbit IgG (H+L) (A21428; Thermo Fisher Scientific, Waltham, MA, USA) or Alexa Fluor 555 goat anti-mouse IgG (H+L) (A21422; Thermo Fisher Scientific) for 2 h at room temperature, followed by washing three times with PBS. The nuclei were stained with DAPI (D9542; Sigma-Aldrich) and slides were mounted in Antifade Mounting Medium (P0126; Beyotime Biotechnology). Images of the mouse colon-tissue sections were acquired using a Leica TCS SP8 high resolution confocal microscope, and images of Caco-2 cells were acquired using a Nikon Eclipse Ni-U fluorescence microscope with NIS-Elements.

### Cell Transfection

293T cells were transiently transfected with pEGFP-N1-SpvB (fusion protein with HA tag) or pEGFP-N1 using ExFect2000 Transfection Reagent (T202; Vazyme, Nanjing, China) for 24 h according to the manufacturer’s instructions.

### Statistical Analysis

Experimental data were analyzed using GraphPad Prism software (La Jolla, CA, USA) and IBM SPSS Statistics for Windows (Armonk, NY, USA). Comparisons between two datasets were performed using the unpaired Student’s *t* test. When comparisons between more than two datasets were performed, the one-way analysis of variance with Bonfferoni *t* test was performed. All data are representative of at least three independent experiments and specific numbers of mice per group are noted in corresponding figure legends. Data for all experiments are presented as the mean ± standard error of the mean (SEM).

## Results

### SpvB Enhances Bacterial Pathogenicity in *S. typhimurium* Infection

**Figure 1:**
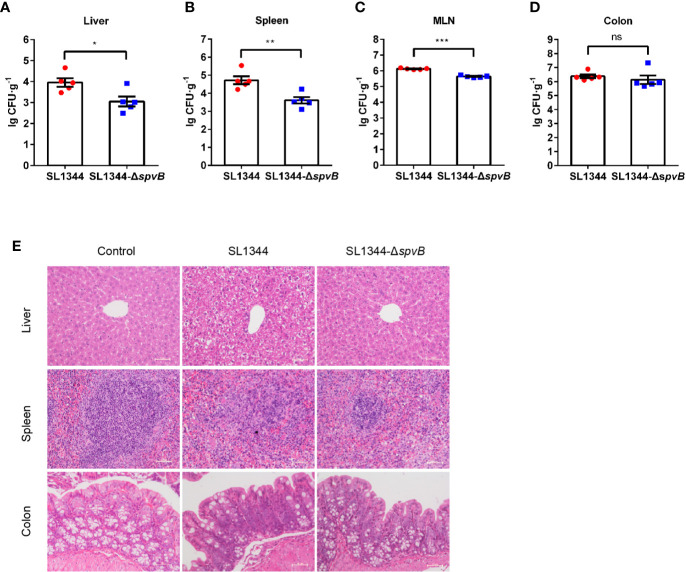
SpvB enhances bacterial pathogenicity in *S. typhimurium* infection. Streptomycin-pretreated mice were orally infected with the indicated *S. typhimurium* strains (1 × 10^8^ CFUs) for 48 h. (A–D) *Salmonella* counts (per gram of the sample) in liver **(A)**, spleen **(B)**, MLN **(C)**, and colon **(D)** of infected mice (n = 5). **(E)** Representative H&E-stained images (scale bar, 50 µm) of liver, spleen and colon tissues from uninfected (control) mice or *Salmonella*-infected mice. Data are presented as the mean ± SEM. ***P < 0.001; **P < 0.01; *P < 0.05; ns, not significant.

We first investigated the effect of the *Salmonella* effector SpvB on bacterial dissemination *in vivo*. *S. typhimurium* wild-type (WT) strain SL1344 or *spvB*-deficient mutant (SL1344-Δ*spvB*) was administered orally with streptomycin-pretreated C57BL/6 mice for 48 h. *Salmonella* counts in liver, spleen and mesenteric lymph node (MLN) of WT strain-infected mice were significantly more than Δ*spvB* strain-infected mice, with no significant difference of bacteria numbers in colon between two infection groups (**Figures 1A–D**). In addition, histopathology in these tissues (liver, spleen and colon) showed more severe pathologic changes in mice infected with WT strain compared with the mice infected with Δ*spvB* strain, including the increasing number of inflammatory cells and impaired cells in liver and spleen, as well as more goblet cell loss and epithelial damage in colon (**Figure 1E**). Together, these results demonstrate that SpvB contributes to *Salmonella* pathogenicity in a mouse model.

### SpvB Contributes to Intestinal Barrier Dysfunction and Bacterial Dissemination *In Vivo*

**Figure 2:**
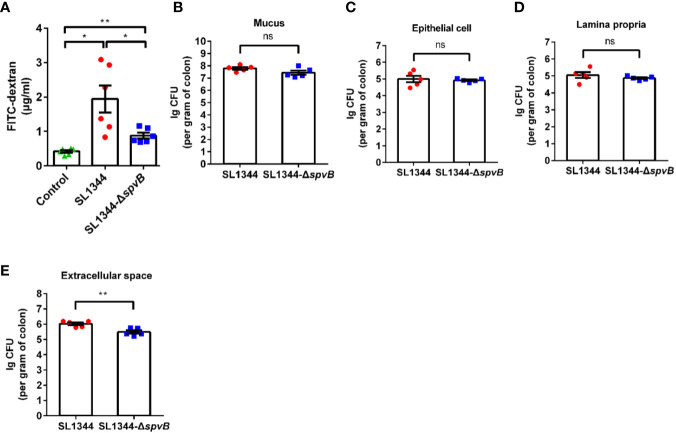
SpvB contributes to intestinal barrier dysfunction and bacterial dissemination *in vivo*. Streptomycin-pretreated mice were orally infected with the indicated *S. typhimurium* strains (1 × 10^8^ CFUs) for 48 h. **(A)** 4 kDa FITC-dextran permeability through the intestinal epithelium of uninfected (control) or *Salmonella*-infected mice in serum. **(B–E)** *Salmonella* counts (per gram of colon) in mucus layer **(B)**, epithelial cells **(C)**, LP cells **(D)** and extracellular space (supernatant fraction, **E**) of the epithelial cell and LP layers from the colon tissue of infected mice (n = 5). Data are presented as the mean ± SEM. **P < 0.01; *P < 0.05; ns, not significant.

Given that the increased bacterial dissemination due to the changed intestinal permeability can be responsible for bacterial pathogenicity, we subsequently analyzed the effect of SpvB on the intestinal permeability *in vivo*. Different groups of *Salmonella*-infected mice were orally administered with the paracellular marker 4 kDa FITC-dextran 4 h prior to sacrifice. Compared with the WT strain-infected mice, the concentration of FITC-dextran in the serum was significantly decreased in Δ*spvB* strain-infected mice (**Figure 2A**). We next enumerated *S. typhimurium* in the mucus layer, epithelial cells, the underlying lamina propria (LP) cells, and extracellular space of the epithelial cell and LP layers from the colon tissue. Relative to the WT strain, the number of Δ*spvB* strain present in the mucus, epithelial cells or LP cells showed no significant changes. However, obviously lower Δ*spvB* counts were observed in the extracellular space of the epithelial cell and LP layers from colon tissue (**Figures 2B–E**). These data suggest that SpvB contributes to the increased intestinal permeability and *S. typhimurium* translocation across the intestinal barrier, thereby enhances bacterial dissemination in mouse colitis models.

### SpvB Contributes to Intestinal Barrier Dysfunction *In Vitro*

**Figure 3:**
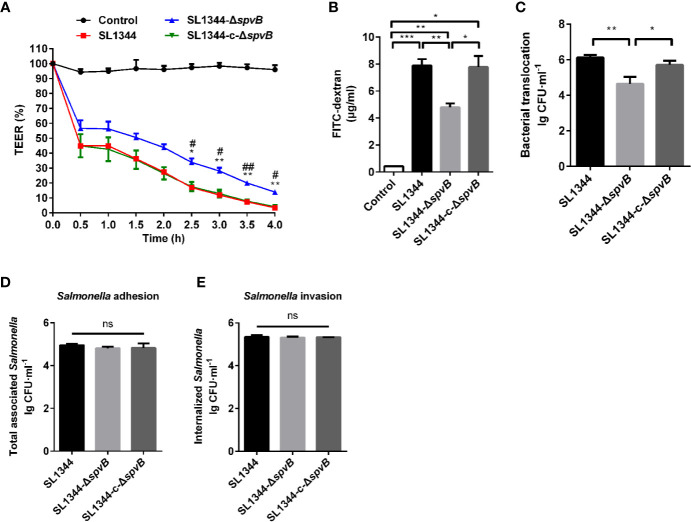
SpvB contributes to intestinal barrier dysfunction *in vitro*. **(A-C)** Caco-2 cells were cultured on Transwell inserts to form cell monolayers, and treated with the indicated *S. typhimurium* strains at an MOI of ~100. TEER **(A)** was measured at the indicated times. Significant difference vs. SL1344 (**P < 0.01; *P < 0.05). Significant difference vs. SL1344-c-Δ*spvB* (^##^*P* < 0.01; ^#^*P* < 0.05). 4 kDa FITC-dextran flux **(B)** and bacterial translocation **(C)** across Caco-2 monolayers were measured 3 h.p.i. **(D, E)** Caco-2 cells were infected with the indicated *S. typhimurium* strains at an MOI of ~100. Total number of associated *Salmonella* (**D**, *Salmonella* adhesion) and the number of internalized *Salmonella* (**F**, *Salmonella* invasion) were determined. Data are presented as the mean ± SEM. ***P < 0.001; **P < 0.01; *P < 0.05; ns, not significant.

We further investigated the effect of SpvB on the intestinal permeability using Caco-2 monolayers, a widely used *in vitro* model to mimic the human intestinal epithelium. As shown in **Figure 3A**, there was a dramatic decrease in transepithelial electrical resistance (TEER) from 0 to 4 h after *S. typhimurium* infection, and cells infected by Δ*spvB* strain exhibited a less decrease than cells infected by strains carrying *spvB*. Furthermore, the less FITC-dextran flux of the Δ*spvB* strain also correlated with the attenuated translocation across Caco-2 cells infected by Δ*spvB* strain compared with the cells infected by strains carrying *spvB* at 3 h post infection (p.i.) (**Figures 3B, C**). Importantly, the translocation defect observed by the Δ*spvB* strain had little connection with the change of bacterial adhesion or bacterial invasion (**Figures 3D, E**). Collectively, these data demonstrate that the SpvB increases epithelial permeability and contributes to intestinal barrier dysfunction both in mouse colitis and Caco-2 monolayer models.

### SpvB Induces Junctional Protein Dysregulation in *Salmonella* Infection

**Figure 4:**
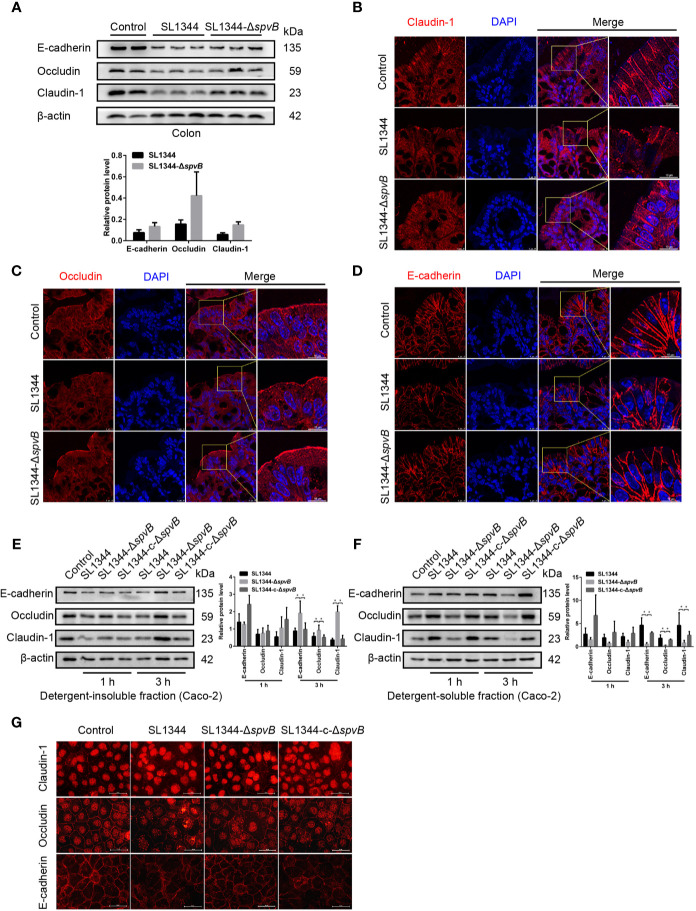
SpvB induces junctional protein dysregulation in *Salmonella* infection. **(A–D)** Streptomycin-pretreated mice were orally infected with the indicated *S. typhimurium* strains (1 × 10^8^ CFUs) for 48 h. **(A)** Western blot analysis and densitometry plots of AJC proteins of colonic IECs from infected mice. **(B–D)** Images of the mouse colon tissue sections showing localization of Claudin-1 (B, red), Occludin (C, red) and E-cadherin (D, red). Nuclei, blue. Scale bar, 10 µm. **(E–G)** Caco-2 cells were treated with the indicated *S. typhimurium* strains at an MOI of ~100. Western blot analysis and densitometry plots of AJC proteins from the detergent-insoluble fraction (E, membrane) and detergent-soluble fraction (F, cytosolic) of Caco-2 cells 1 h and 3 h.p.i. **(G)** Images of Caco-2 cells showing membrane localization of Claudin-1 (red), Occludin (red) and E-cadherin (red) 3 h.p.i. Scale bar, 50 µm. Data are presented as the mean ± SEM. *P < 0.05.

AJC, including TJ and AJ, plays a crucial role in the maintenance of intestinal paracellular permeability. Thus, we examined the expression and distribution of TJ proteins Claudin-1 and Occludin, and AJ protein E-cadherin in the colon tissue of *Salmonella*-infected mice. Western blot analysis of colonic IECs of mice infected with Δ*spvB* strain for 48 h showed slightly reduced Claudin-1, Occludin and E-cadherin levels compared with WT strain-infected mice (**Figure 4A**). Immunostaining of the colon tissue sections revealed the greater disruption of Claudin-1, Occludin and E-cadherin in IECs of mice challenged with WT strain, compared with Δ*spvB* strain (**Figures 4B–D**). Next, we analyzed the above cell junction proteins in the detergent-insoluble and -soluble fractions of Caco-2 cells. Relative to the strains carrying *spvB*, the Δ*spvB* strain exhibited significantly increased Claudin-1, Occludin, and E-cadherin levels in the detergent-insoluble fractions at 3 h p.i. While in the detergent-soluble fractions, a decreased level of these proteins at 3 h p.i. was observed (**Figures 4E, F**). Similarly, immunostaining of the Caco-2 cells confirmed more severe membrane mislocalization of Claudin-1, Occludin, and E-cadherin after infection with the strains carrying *spvB* at 3 h p.i. (**Figure 4G**). Taken together, these data indicate SpvB induces junctional protein (Claudin-1, Occludin, and E-cadherin) dysregulation in *Salmonella*-infected mice, and contributes to the subcellular redistribution of these proteins in Caco-2 cells.

### F-Actin Rearrangement Is Associated With SpvB-Mediated Barrier Dysfunction

**Figure 5:**
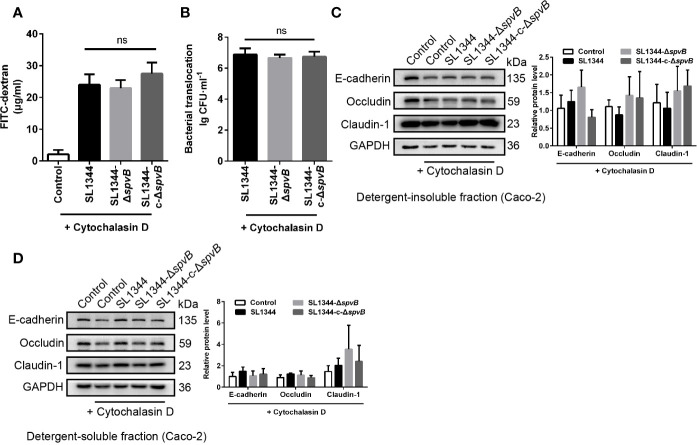
F-actin rearrangement is responsible for SpvB-mediated barrier dysfunction. Caco-2 cells were pre-treated with the inhibitor of actin polymerization, Cytochalasin D (2 µM), 1 h prior to infection with the indicated *S. typhimurium* strains. 4 kDa FITC-dextran flux **(A)** and bacterial translocation **(B)** across Caco-2 monolayers were measured 3 h.p.i. **(C, D)** Western blot analysis and densitometry plots of AJC proteins from the detergent-insoluble fraction (C, membrane) and detergent-soluble fraction (D, cytosolic) of Caco-2 cells 3 h.p.i. Data are presented as the mean ± SEM. ns, not significant.

Previous study of SpvB has shown its potential function in depolymerizing actin filaments (Tezcan-Merdol et al., 2001). To determine whether F-actin rearrangement is involved in SpvB-mediated barrier dysfunction, we pretreated Caco-2 cells with the actin polymerization inhibitor Cytochalasin D. As shown in **Figures 5A, B**, Cytochalasin D markedly increased the FITC-dextran flux and *Salmonella* translocation in all of the Caco-2 cell infection groups, and importantly, Cytochalasin D eliminated the differences among these infection groups. Moreover, we measured the cell junction proteins in the detergent-insoluble and -soluble fractions from Caco-2 cells after Cytochalasin D treatment. The western blot analysis showed there were no significant differences among Claudin-1, Occludin and E-cadherin expression in either the detergent-insoluble or -soluble fraction from Caco-2 cells (**Figures 5C, D**). Therefore, these results suggest that F-actin rearrangement is responsible for SpvB-mediated barrier dysfunction in *Salmonella*-infected Caco-2 cells.

### Down-Regulated PKC Activity Is Involved in SpvB-Mediated Barrier Dysfunction

**Figure 6:**
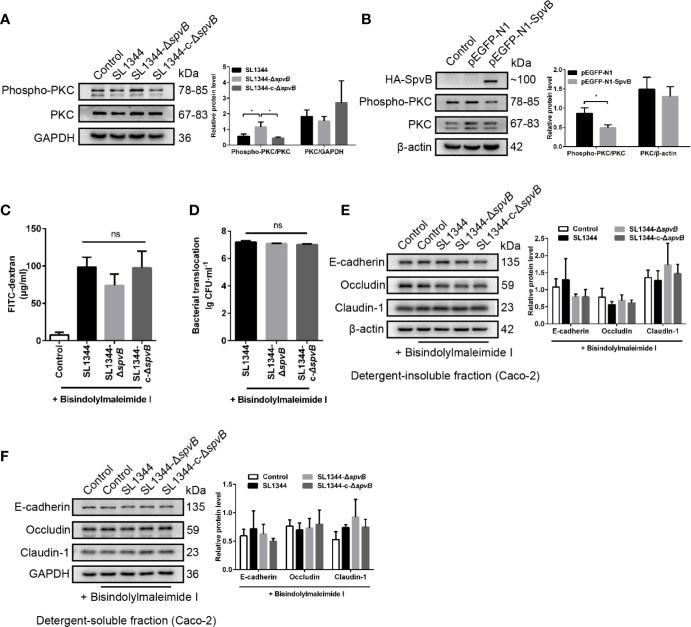
Down-regulated PKC activity is involved in SpvB-mediated barrier dysfunction. **(A)** Caco-2 cells were treated with the indicated *S. typhimurium* strains at an MOI of ~100. Western blot analysis and densitometry plots showing the activation of PKC. **(B)** 293T cells were transfected with pEGFP-N1-SpvB or pEGFP-N1 for 24 h. Western blot analysis showing the expression of HA-SpvB and the activation of PKC. **(C–F)** Caco-2 cells were pre-treated with the inhibitor of PKC activation, Bisindolylmaleimide I (1 µM), 1 h prior to infection with the indicated *S. typhimurium* strains. 4 kDa FITC-dextran flux **(C)** and bacterial translocation **(D)** across Caco-2 monolayers were measured 3 h.p.i. **(E, F)** Western blot analysis and densitometry plots of AJC proteins from the detergent-insoluble fraction (E, membrane) and detergent-soluble fraction (F, cytosolic) of Caco-2 cells 3 h.p.i. Data are presented as the mean ± SEM. *P < 0.05; ns, not significant.

PKC signaling pathway has been reported to regulate paracellular permeability *via* cytoskeleton rearrangement and modulates cell junction protein expression (Corr et al., 2014; Fan et al., 2018). We observed that SpvB resulted in a decrease in PKC activity in Caco-2 cells at 3 h p.i. using western blot analysis (**Figure 6A**). To investigate whether the inhibition of PKC activity is directly due to the effect of SpvB, pEGFP-N1-SpvB plasmid was transfected into 293T cells for ectopic expression. Western blot analysis confirmed the expression of HA-tagged fusion SpvB, and the decreased PKC activity in pEGFP-N1-SpvB plasmid transfection group (**Figure 6B**). To further confirm the involvement of PKC activation in SpvB-mediated barrier dysfunction, we pretreated Caco-2 cells with Bisindolylmaleimide I, a PKC inhibitor. As shown in **Figures 6C, D**, Bisindolylmaleimide I increased the FITC-dextran flux and *Salmonella* translocation, and all infection groups showed no significant differences in statistics. Similarly, the western blot exhibited no apparent differences in the cell junction proteins after Bisindolylmaleimide I treatment, no matter in the detergent-insoluble or -soluble fraction from Caco-2 cells (**Figures 6E, F**). Together, these results support our hypothesis that the inhibition of PKC activity is involved in SpvB-mediated barrier dysfunction following *Salmonella* infection *in vitro*.

## Discussion

*Salmonella* are typical foodborne pathogens and have emerged as a major global public health concern. After oral infection, non-typhoidal *Salmonella* usually remain localized to intestinal tissues for survival and replication, and sometimes manage to pass through intestinal barrier for systemic spread *via* both transcellular and paracellular pathways. As an effective physical barrier against enteric pathogens, intestinal epithelial barrier plays a key role in regulating the localized or systemic infection of non-typhoidal *Salmonella* (Hu et al., 2016; Pardo-Roa et al., 2019). As expected, *Salmonella* have evolved intricate strategies to overcome or manipulate mechanisms of the intestinal epithelial barrier integrity. Previous studies have identified that a few bacterial effectors secreted by type three secretion system (T3SS) are associated with intestinal epithelial barrier dysfunction in *Salmonella* infection (Boyle et al., 2006; Lin et al., 2016). Nevertheless, more knowledges of effectors encoded apart from bacterial chromosomes remain to be explored. The effector SpvB is encoded by bacterial virulence plasmid existing extensively in clinical important *Salmonella* serovars, and released into the host cell through *Salmonella* pathogenicity island-2 (SPI-2) T3SS. Here, we used a mouse model and a human Caco-2 cell model of *S. typhimurium* infection. C57BL/6 mice were orally challenged with *S. typhimurium* for 48 h, and we first determined bacterial numbers and tissue damage of major organs, so as to emphasize the role of SpvB in the pathogenesis of *S. typhimurium*. Bacterial dissemination caused by intestinal barrier dysfunction plays a key role in determining the outcomes of infection with enteric pathogens, and we further evaluated the intestinal permeability. Using the above mouse model, we report that SpvB contributes to translocation of *S. typhimurium* from the intestinal lumen across the gut epithelium, and subsequent systemic dissemination. In addition, we used *S. typhimurium*-infected Caco-2 cell model to show that SpvB impairs the intestinal epithelial barrier integrity in both models, which demonstrated the contribution of SpvB to bacterial pathogenicity. In general, the study uncovers the relationship between SpvB and intestinal epithelial barrier in *S. typhimurium* infection, which broadens our understanding of virulence factors utilized by *Salmonella* to spread to the extraintestinal organs like liver and spleen.

The paracellular route allowing the translocation of bacteria and their production is mainly regulated by AJC including TJ and AJ. They are complex protein structures comprised of transmembrane proteins such as Claudin-1, Occludin, and E-cadherin, which interact with the actin cytoskeleton *via* plaque proteins. Bacterial effectors that contribute to changes of intestinal epithelial barrier integrity usually cause one or more of these AJC transmembrane proteins redistribution (Drolia et al., 2018). Here, we observed the lower levels and more redistribution of major TJ proteins Claudin-1 and Occludin, and AJ protein E-cadherin caused by SpvB in mouse colonic IECs, suggesting SpvB could affect intestinal epithelial barrier integrity through redistributing these AJC transmembrane proteins. Then we further separated Caco-2 cell membrane and cytoplasmic protein, and found lower levels of above AJC proteins in the membrane of cells result from SpvB, accompanied by more severe protein redistribution. Our observation *in vivo* and *in vitro* indicates that SpvB promotes AJC proteins disassembly and redistribution by some ways during *S. typhimurium* infection, providing more evidence in SpvB-mediated paracellular translocation of *Salmonella*.

To explore the underlying mechanism involved in SpvB-mediated intestinal barrier dysfunction, we first investigated from the perspective of SpvB. There are two distinct domains separated by a run of several proline residues in SpvB. It has been reported that the C-terminal domain of SpvB contains ADP-ribosyl transferase activity, which modifies G-actin monomers and prevents their polymerization into F-actin filaments (Lesnick et al., 2001). Considering that F-actin is indispensable in maintenance of AJC, we used F-actin inhibitor Cytochalasin D to confirm that depolymerization of F-actin is involved in SpvB-mediated intestinal barrier dysfunction. So far, SpvB has been proved to increase cell damages mainly through its F-actin depolymerization-associated function, which was studied in macrophages like J774.1 and RAW264.7 cell lines, and epithelial cells like HeLa cell line (Buckner et al., 2011; Chu et al., 2016; Yang et al., 2019). In this study, we bridge *Salmonella* effector SpvB and intestinal epithelial cells, which serve as a portal of entry for enteric pathogens, and showed that SpvB-mediated barrier dysfunction is associated with the regulation of F-actin rearrangement, which is consistent with previous studies on other cell types.

In addition to the direct regulation of cytoskeleton, there are signaling pathways involved in the assembly, disassembly and maintenance of AJC. They are controlled by a number of signaling molecules, such as PKC, mitogen-activated protein kinases, myosin light chain kinase, and Rho GTPases (Turner, 2009). PKC is a family of serine-threonine kinases, and it is known that activation of PKC is related to enhanced barrier function (Suzuki et al., 2009; Corr et al., 2014). Interestingly, we have found the inhibition of PKC activity result from SpvB by western blot analysis, and further confirmed the inhibition using pEGFP-N1-SpvB plasmid. Previous studies have reported that bacterial effectors like zonula occludens toxin from *Vibrio cholerae* and OspE proteins from *Shigella flexneri*, could target PKC to modulates diverse cellular responses including barrier function (Fasano et al., 1995; Yi et al., 2014). In this study, we subsequently used PKC inhibitor Bisindolylmaleimide I in all infection groups to further evaluate PKC’s role in barrier function, and the results support our hypothesis that inhibition of PKC was involved in SpvB-mediated intestinal barrier dysfunction. The mechanism concerning how SpvB acts with PKC remains further investigation.

**Figure 7:**
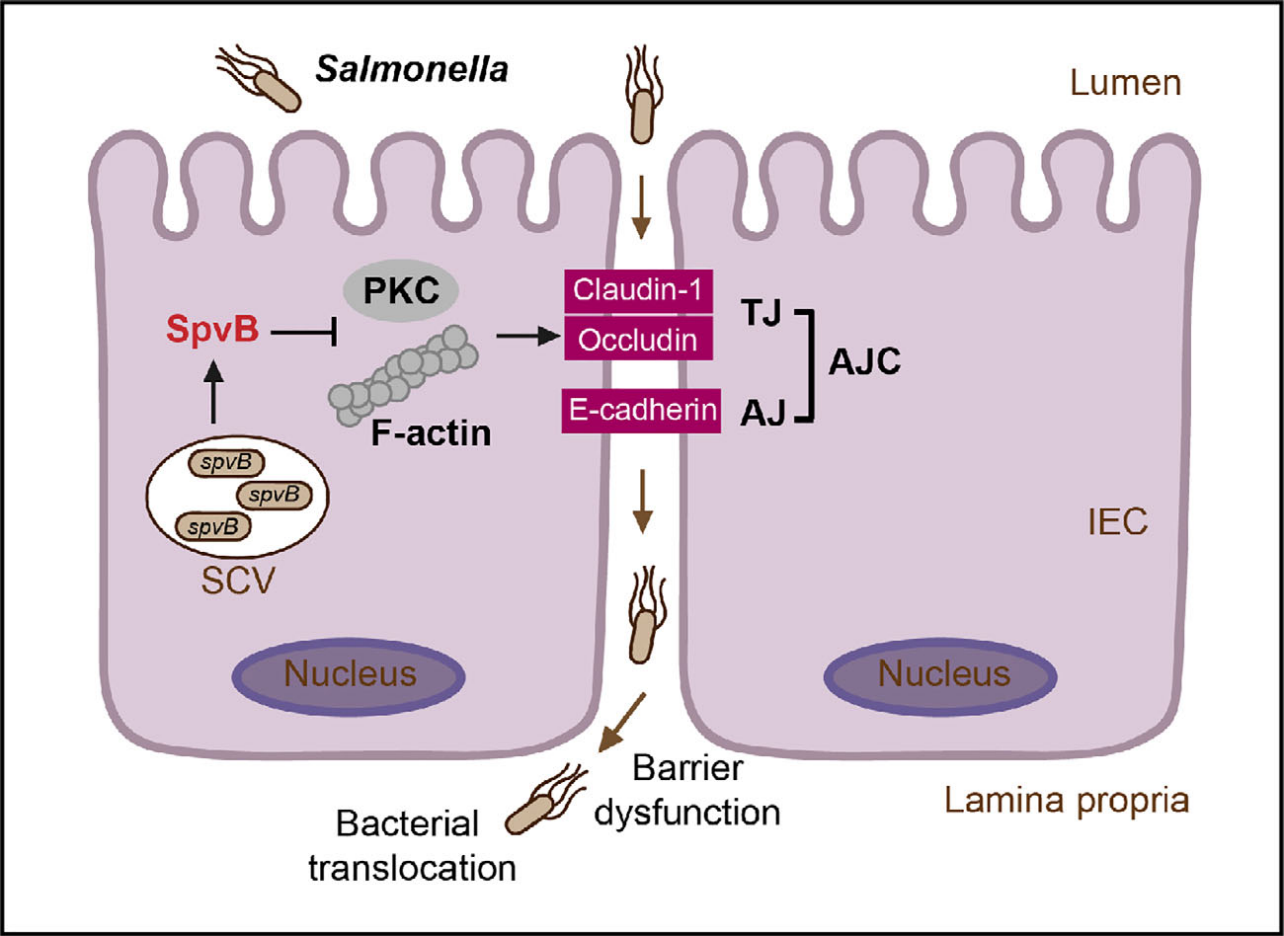
General scheme of SpvB-mediated bacterial translocation during *Salmonella* infection. *Salmonella* effector SpvB released into the intestinal epithelial cell could lead to F-actin rearrangement and suppression of PKC signaling pathway, which contributes to the cellular redistribution of Claudin-1, Occludin and E-cadherin junctional proteins. This SpvB-mediated dysfunction of intestinal epithelial barrier integrity thereby facilitates bacterial translocation *via* the paracellular route to promote *Salmonella* systemic dissemination. AJ, adherens junction; AJC, apical junctional complex; IEC, intestinal epithelial cells; PKC, protein kinase C; SCV, *Salmonella*-containing vacuole; TJ, tight junction.

Taken together, our study connects *Salmonella* effector SpvB to intestinal epithelial barrier, not only revealing a novel contribution of SpvB to pathogenesis of *Salmonella* by disrupting intestinal epithelial barrier integrity, but also uncovering *Salmonella*-induced intestinal barrier dysfunction from the perspective of an important effector encoded in *Salmonella* virulence plasmid. During *S. typhimurium* infection, SpvB inhibits PKC signaling pathway and exerts its regulation of F-actin to redistribute junctional proteins like Claudin-1, Occludin, and E-cadherin, thereby facilitate bacterial translocation across the gut epithelium and increase bacterial dissemination (**Figure 7**). Our findings broaden the understanding of virulence factors utilized by *Salmonella* in bacterial paracellular translocation, and provide new strategies for the therapy in limiting the systemic spread of bacteria during infection.

## Data Availability Statement

The raw data supporting the conclusions of this article will be made available by the authors, without undue reservation.

## Ethics Statement

The animal study was reviewed and approved by the Ethic Committee of Soochow University.

## Author Contributions

LS, SY, and RH designed the experiments. LS, SY, QD, and KD performed experiments and wrote the manuscript. YL, SW, and RH supervised the project and edited the manuscript. All authors contributed to the article and approved the submitted version.

## Funding

This work was supported by Natural Science Foundation of China (No. 81971899, No. 31970132, No. 81671976, No. 31670140), a project funded by the Priority Academic Program Development (PAPD) of Jiangsu Higher Education Institutions and Suzhou Municipal Science and Technology Foundation (SYS2019031).

## Conflict of Interest

The authors declare that the research was conducted in the absence of any commercial or financial relationships that could be construed as a potential conflict of interest.

## Abbreviations

AJ, adherens junction; AJC, apical junctional complex; BSA, bovine serum albumin; CFUs, colony forming units; DMEM, Dulbecco’s Modified Eagle’s medium; FBS, fetal bovine serum; H&E, hematoxylin and eosin; IECs, intestinal epithelial cells; LB, Luria Bertani; LP, lamina propria; MLN, mesenteric lymph node; MOI, multiplicity of infection; p.i., post infection; PKC, protein kinase C; PVDF, polyvinylidene difluoride; *S. typhimurium*, *Salmonella enterica* serovar Typhimurium; SCV, *Salmonella*-containing vacuole; SEM, standard error of the mean; SPI-2, *Salmonella* pathogenicity island-2; *spv*, *Salmonella* plasmid virulence; T3SS, type three secretion system; TBS, Tris-buffered saline; TBST, Tris-buffered saline containing 0.1% Tween 20; TEER, transepithelial electrical resistance; TJ, tight junction.
